# Autonomous, miniature research station (lab-payload) for the nanosatellite biological mission: LabSat

**DOI:** 10.1038/s41598-025-16044-y

**Published:** 2025-08-22

**Authors:** Patrycja Śniadek, Agnieszka Krakos, Adrianna Graja, Bartosz Kawa, Rafał Walczak, Jan Dziuban

**Affiliations:** Division of Microsystems, Faculty of Electronics, Photonics and Microsystems, Wroclaw University of Science and Technology, 27 Wybrzeze Wyspianskiego Street, 50-370 Wroclaw, Poland

**Keywords:** Lab-payload, Lab-chip, Bio-nanosatellite, Microfluidic, Astrobiology, Plant sciences, Engineering, Aerospace engineering, Techniques and instrumentation

## Abstract

There is an increase in demand for bio-nanosatellites and biomedical methodologies as a result of experiments conducted in microgravity and radiation conditions. Currently, the latest trend is to replace the experiments carried out by cosmonauts at the International Space Station (ISS) with research performed with the use of autonomous payload for nanosatellite. This paper describes the lab-payload for a biological nanosatellite of the CubeSat type with a size of 2U (10 × 10 × 20 cm^3^). The proposed payload enables the long-term cultivation of two different biological experiments simultaneously and provides suitable growth conditions. This lab-payload is equipped with lab-chips dedicated to each of the cultures, a container with a nutrient solution, a medium dosing system, an optical detection system, lighting, a heating system and sensors for measuring temperature, humidity, pressure and radiation inside a thermos.

## Introduction

The influence of space environments on a widely understood life, especially in the context of microgravity and radiation, is one of the most interesting subjects recently raised by researchers. In the previous, long-term work on attempts to describe and eliminate the effects of the impact of the space environment on living objects, an important source of data was observations and experiments conducted as part of manned missions. Due to ethical, economic, and organizational aspects, research methods using so-called model organisms are employed, whose similarities with cells and functional processes in the human body are well known^1^. Additionally, rapid growth and short life cycles allow for efficient assessment of the space environment impact at various stages of development and over several generations. The use of small, model plants in research has similar advantages. Additionally, the same reasons why plants are necessary to sustain life on Earth (including food production, CO_2_ absorption, release of O_2_ and water vapor) make them an important element in the construction of space habitats and the implementation of long-term manned missions^1^.

Microbiology research in space falls into two broad categories that connect biological sciences with space: astrobiology and basic space biology. Most of this research, in its initial stage, is performed using devices that simulate microgravity on Earth (RPM – Random Positioning Machine^2^, RWV – Rotary Wall Vessel^3^). Therefore, testing on a simulator may be an intermediate step in targeted research (validation equipment) or may be helpful in the interpretation of results obtained in the space environment (reference test)^4^. However, neither the microgravity environment nor the complex, natural spectrum of space radiation can be recreated or effectively simulated in terrestrial laboratories. Comprehensive research concerning the impact of these factors on living organisms can only be conducted in space, using infrastructure that provides them with basic support for life processes (e.g. atmosphere, nutrition, temperature control).

An important role in space research is played by the International Space Station—ISS, which has been pursuing various research goals since 1998^5^. Approximately 2,700 different scientific studies have been carried out on board, including several hundred related to biology, medicine, and biotechnology. Depending on the requirements of the experiment to be conducted, power supply, support for acquisition and telemetry of measurement data, appropriate thermal conditions, or supply/discharge of gas mixtures can be provided^6^. Most importantly, in the case of research on biological samples, it is possible to deliver them to the station, e.g. in frozen form, and recover them for more detailed analysis using terrestrial equipment. This is possible thanks to the professional handling of experiments by astronauts. However, providing such a wide range of services generates high costs for a single experiment. There are also problems with complicated regulations, complex planning requirements, and long waiting times for an experiment due to the high demand for such research. As a response, an interesting commercial opportunity has been recently provided—ICE Cube ISS facility. It offers a modular, plug-and-play system for conducting experiments in microgravity. Apart from the assistance during experiment development, it is possible to prepare the samples and integrate the whole structure as close to the launch as possible. At Space Applications Services, ICE Cube facility also provides its own mission control software—YAMCS, which is available for continuous experiment monitoring.

Biological experiments in microgravity can also be conducted on the upper stage of rockets, very often after the completion of the prime mission. One example is the PSLV structure from India, which in 2019 showed its successful demonstration with three individual payloads on board. Larger rockets can also be applied for biological experimentation, but typically they are used to study the effects of harsh space environments in the context of microgravity and radiation, in deep space missions. Apart from BioSentinel, for example, BioExpt-01 can be mentioned, which is planned during the Artemis mission. Its main goal will be to investigate the impact of space flight beyond the Van Allen radiation belt onto amino acids in *Arabidopsis thaliana* seeds. As shown, the development and adaptation of other solutions to conduct biomedical research in space can be observed. Taking into account a great emphasis placed on reducing the costs of launching experiments into orbit, the use of small satellite structures in the micro (10–100 kg), nano (1–10 kg), or pico (0.1- 1 kg) size^7^ is becoming increasingly common. Currently, the CubeSat standard is commonly used, the basic unit of which is a cube with a side of 10 cm (1U), duplicated depending on the needs. CubeSat has gained great popularity thanks to such features as miniaturization, standardized dimensions, and procedures for testing and integration with a rocket, a known method of placing them in a selected orbit, and their disposal by burning in the atmosphere^8,9^.

Similarly to the general approach to the construction of satellites, the construction of a CubeSat can be divided into two main parts. The first part is a satellite platform for supporting structure with standardized dimensions along with electronic modules (e.g. power supply, communication) that perform basic satellite functions. The second part is a payload, a system that performs a task that is usually the main goal of a satellite mission.

Thanks to the use of the aforementioned satellite solutions, small scale satellites used to conduct biological research, known as bio-satellites, are currently undergoing rapid development. Most of the conducted experiments used optical detection methods, including fluorescence, colorimetry, and image acquisition. It is also worth noting that almost all of the CubeSat bio-satellites used lab-chip systems to carry out experiments. Only 11 CubeSat bio-satellites have been launched so far (Table 1).

**Table 1:** List of biological satellites using the cubeSat standard.

Mission name	Satellite size (payload)	Aim of the experiment	Detection system	Year	Institution	Time of experiment	Integration time	Microfluidic platform/ lab-chip material	Satellite photo
GeneSat-1	3U (2U)	Cultivation of *E. coli* bacteria with simultaneous characterization of their growth and metabolism	fluorescence	2006	NASA	~ 100 h	5 weeks	Acrylic lab-chip card ^10^	
									^10^
PharmaSat	3U (2U)	the influence of microgravity on the growth and metabolism of yeast (*S. cerevisiae*) with simultaneous testing of the effectiveness of antifungal agents	Optical density changes and colorimetry	2009	NASA	96 h	6 weeks	poly(methylmethacrylate) fluidic card^11^	
									^11^
O/OREOS	3U (2 × 1U)	Assessment of the stability of organic compounds	Spectroscopy	2010	NASA	6 months	–		
								SEVO stainless steel spacer ring with sapphire and MgF_2_ glass windows^13^	^12^
		Characterization of the growth and metabolism of *B. subtilis* and *H. chaoviatoris* bacteria							
								SESLO ^12^	
SporeSat	3U (2U)	Investigate the effect of gravity on the reproductive spores of the fern, *Ceratopteris richardii*	Measurement of calcium ion concentration with ion-selective electrodes	2014	NASA	4 days	–	Fused silica disc^14^	^15^
EcAMSat	6U (3U)	Characterization of the growth and metabolism of *E. coli* bacteria and the effectiveness of antibiotics	Optical density changes and colorimetry	2017	NASA	156,5 h	8 weeks	Polymethylmethacrylate (PMMA) fluidic card^16^	
									^17^
DIDO-2/3	3U (2U)	Study the effect of microgravity and low fluid shear with four different experiments on biological material	DIDO-3 – Spectrophotometry	2017 (DIDO-2) 2020 (DIDO-3)	SpacePharma	DIDO-3 – 40 days	–	Fluidic card – no information^18^	
									^19^
Biosentinel	6U (4U)	Research of DNA damage in the yeast *S. cerevisiae*	colorimetry, measurement of optical density, measurement of energy and radiation dose with a LET spectrometer	2022	NASA	6–12 months	–	fused polycarbonate (PC) fluidic card^20^	
									^21^
PlantSat	3U (1U)	Research of extremophiles and plants (*Tillandsia*)	–	2022	University of Chile	–	–	–	
									^22^
GreenCube	3U (2U)	Cultivating micro greens (*Lepidium sativum)*	Optical detection (micro-camera)	2022	Sapienza University of Rome	20 days	–		
								^24^	^23^
AstroBio	3U (1,5U)	Immunoassay tests	Chemiluminescence detection	2022	Sapienza University of Rome	6 experiments in 10 min each	–	Origami microfluidic – chromatographic paper^26^	
									^25^

In 2022, as many as 4 satellites with biological missions were launched, which demonstrates that this topic is being significantly developed. The dominant trend in the construction of payloads for conducting biological research in space is miniaturization. Most of the described missions used lab-chip technology and optical detection methods to analyze the results of individual experiments. Most microfluidic cards are made using polymer technology. So far, fused silica and chromatographic paper have only been used once to construct nanosatellite microfluidic platforms. To date, three attempts to conduct experiments using *E.coli* bacteria have been made. Plants have also been used for research purposes three times, of which it is known that the SporeSat mission failed, and the results of the remaining two are unknown. When further comparisons are made, it can be seen that only the O/OREOS satellite contained two radically different biological experiments that required the use of different microfluidic structures. It should also be noted that three of the afromentioned satellites attempted to carry out experiments outside LEO (BioSentinel, GreenCube, and AstroBio), which may indicate a new trend in research. Many of the above missions faced various problems, such as damage to the dosing system or lighting, which rendered them unsuccessful. However, an often-mentioned problem is the need to wait a long time for the launch of the rocket carrying the satellite, which is critical in the context of biological assays^27^. As reported in the paper^28^, integration of the payload typically occurs approximately circa 1–3 months prior to the launch. CubeSats are then stored in the hangar, where the temperature is rather ambient. Moreover, during this phase, the payload cannot be operated, thus life-support systems do not work. Consequently, highly sensitive samples, e.g. mammalian cells, that require elevated incubation conditions (37–39 °C) and culturing medium perfusion, are not the first choice for the CubeSat missions. Thus, model microorganisms (e.g. bacteria, fungi) which can be prepared in a dehydrated form have been typically used for this purpose so far. So-called “late access” options are available, which can shorten the waiting times notably, nevertheless, they do not guarantee that some unexpected shifts (e.g. weather) will not occur and the launch will be postponed. All these factors make this issue very complex and therefore, a niche area. In response to this problem, recent studies have raised the possibility of long-term cancer cell cultures provided in ambient temperature and without supply of the fresh media have been raised in the literature recently^28^. Authors showed that circa 54 days of culturing in the aforementioned conditions is possible, but still, it may not comply with launch constraints.

Despite many difficulties in the construction of CubeSat bio-satellites, the further development of this type of laboratory is very important due to a number of advantages, including the lack of human operational input and access to various research environments (selection of orbit). Simultaneously, looking at the ongoing works carried out on interplanetary missions, the development of a wide range of payloads enabling various biological research is very desirable.

This article describes a new fully autonomous laboratory platform that enables space research on plant and fungi samples. This is the first nanosatellite payload combining such diverse biological experiments (fungi and seed) to be conducted in LEO. It uses lab-chips made in glass and for the first time 3D printing technology. An optical detection system is proposed in the form of a miniature CMOS camera with adjustable focus to control the growth of individual biological objects. The payload structure includes heaters that enable the maintenance of the correct temperature for the selected experiments, lighting, and a medium dosing system. The entire laboratory is closed in a tight thermos ensuring atmospheric pressure. Details on research methodology, design, and payload fabrication, as well as mission results, are described thoroughly in the following sections of this paper.

## Materials and methods

The aim of the mission of the LabSat bio-nanosatellite was to evaluate the operation of the newly developed lab-payload enabling biological research, connected to the satellite platform provided by SatRev company (Wroclaw, Poland). The entire bio-nanosatellite was 3U in size, of which the payload took 2U space. The success criterion of the presented mission was the verification of the proper functioning of each of the subsystems discussed below, all of which enable the execution of biological experiments. These included: glass and 3D-printed lab-chip devices, the optical detection system, the culture medium dispensing system, maintenance of appropriate temperatures for each culture, and the airtightness of the custom-designed thermos. The verification tests were related to our substantial experience in developing fully-featured ground-based biomedical platforms, implementation of simulated microgravity experiments (RWV, RPM), and tests directly qualifying for the launch.

From a biological standpoint, the mission aimed to assess the feasibility of long-term storage of biological samples within lab-chip platforms. Additionally, it sought to confirm previously observed results regarding the specific development of biological specimens under simulated microgravity conditions on Earth—namely, the enhanced growth of fungal mycelium and the slower, non-specific germination of seeds due to altered geotropism responses.

**Fig. 1:**
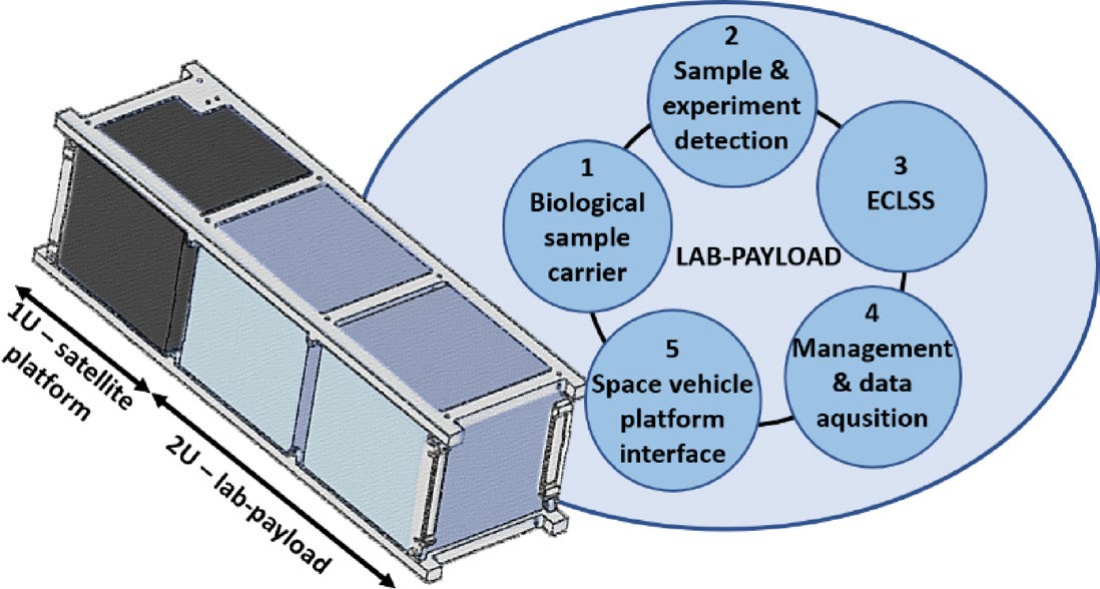
Conception of LabSat bio-nanosatellite with five basic functional blocks of lab-payload to conduct a biological experiment.

Based on a review of previous biological missions and preliminary requirements, a lab-payload implementation scheme was developed to enable biological research on the satellite platform. Five main functional components have been identified (Fig. 1).

As presented in Fig. 1 the first of the elements is the biological sample carrier. It protects the biological object, enabling its cultivation. The second element is the detection system that provides data to assess the condition of the object and the course of the experiment. The third element, ECLSS (Environmental Control and Life Support System)—is responsible for control and support, i.e. providing cooperating sensors and actuators responsible for monitoring and/or controlling environmental conditions, including temperature and medium supply/discharge. The fourth element is the experiment management and data acquisition system, implemented as an electronic system with built-in software. The last element is the lab-payload interface with the satellite platform, i.e. electrical and signal cabling with the implementation of a communication bus. It is responsible for supplying power to the lab-payload and exchanging commands and data with the satellite platform modules. The implementation of each element will be described in the following sections of the article below.

### Boundary conditions for biological experiments

**Fig. 2:**
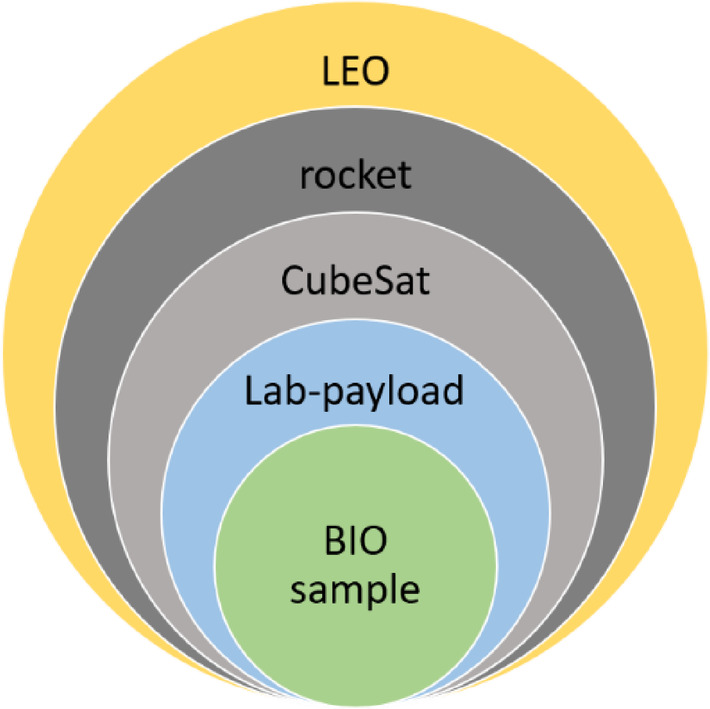
Sources of requirements for implementing the CubeSat biological payload in LEO.

In our analysis, the lab-payload design process was divided into “layers” (Fig. 2). The analysis of the requirements for the correct operation of the biological payload began with the limitations resulting from the environment in which the experiment is to be carried out, i.e. LEO. The guidelines imposed by the nanosatellite launch service provider, the standard CubeSat platform provider, and the conditions resulting from conducting biological research were further defined. A summary of the requirements for biological payload is presented in Table 2.

**Table 2:** Detailed analysis of requirements.

	Lab-payload requirements	Comment
LEO	Lab—payload remains functional during the satellite’s exposure to particle radiation and protects the biological object from its influence	The value of the accumulated radiation dose depends, among others, on the time and orbit parameters and the method of implementing the construction of the lab—payload and satellite platform
	Lab—payload remains functional when the satellite is exposed to variable heat fluxes	Operating temperature range for the satellite’s internal components: from − 40 °C to + 50 °C^29^
	Lab—payload remains functional during the vacuum exposure	The range of changes from 10^–6^ to 10^–4^ Pa^30^
	Lab—payload remains functional in microgravity conditions	The range of changes from 10^–6^ to 10^–3^ m/s^2^^30^
ROCKET	Materials used for the construction of the lab-payload (especially the outer casing and mounting elements) are characterized by very low gassing coefficients in vacuum	Total Mass Loss (TML) ≤ 1.0% Collected Volatile Condensable Material (CVCM) ≤ 0.1% According to 3.1.8.1 and 3.1.8.2^32^
	Materials used for the construction of the lab—payload were not included in the list of hazardous materials	According to the Table 49 CFR §172.10^33^
	Lab—payload fulfill electromagnetic compatibility requirements	Verification required according to launch service provider specifications
	Lab—payload remains functional after lunch acceleration	Verification required in random vibration test, according to launch service provider specifications
	Lab—payload remains functional after the satellite is exposed to the thermal conditions of rocket launch	Verification in a thermal-vacuum test, according to launch service provider specifications
	Biological experiment is adjusted to “wait” time for launch in orbit (pause/off mode)	Shutting down a CubeSat takes about 3–5 months, a biological experiment may be suspended even longer due to the need to carry out the process of assembly, integration, and testing of the device
CUBESAT	Nanosatellite volume – 3U Lab—payload volume ≈ 2U	On the selected 3U satellite platform (SatRev) ≤ 96 mm × 96 mm × 190 mm including mounting elements
	Lab—payload weight ≈ 2.66 kg	Accordance with 3.2.13^30^ taking into account that the center of gravity of the CubeSat should be ≤ 20 mm from its geometric center in the X and Y directions (3.2.14^31^)
	Materials used for the construction of the lab-payload (especially the packaging and mounting elements) do not have ferromagnetic properties	Elimination of a potential source of disturbances in the operation of other satellite modules
	Maximum current consumed by the lab-payload: 3 A	According to the specifications of the universal satellite platform from SatRev: Interface Control Document rev 2.3
	Available voltages for lab-payload operation: 3.3 V, 5 V, 12 V	
	Constant power consumption of lab-payload at 5 V: max. 15 W	
	Lab-payload has its own electronic module for managing the experiment and recording experiment data	The On-Board Computer (OBC) module is an on-board computer only for satellite platform modules
	Exchange of lab-payload data with platform modules using the CAN bus	Transport and network layer defined by CSP (CubeSat Space Protocol)
	Transmission of lab-payload data packets at a speed of ≤ 550 kb/s	Direct addressing to the satellite radio module
LAB-PAY LOAD	Lab-payload maintains the culture gas mixture	The composition of the culturing gas mixture depends on the biological object
	Lab-payload maintains the appropriate temperature of biological samples and culture medium	Plus temperatures, the exact value/range varies depending on the biological object
	Lab-payload ensures the flow of culture medium	Range from µl/min to ml/min, exact values depend on the method of implementing the biological sample carrier and the microfluidic system as well as the biological object being tested
	Lab-payload provides the measurement of the humidity of the indoor atmosphere	Humidity is important for traditional culturing; the range required varies depending on the biological object
	Lab-payload protects against cosmic radiation	Lab-payload is intended to enable the assessment of the impact of microgravity on a biological object
	Lab—payload ensures the detection of the biological sample and the progress of the experiment	Required non-invasive method that does not affect the condition of the biological object or the progress of the experiment

#### Biological samples

Due to the long waiting time for the rocket launch, it was decided to prepare the experiments with spores of fungi from the *Fusarium culmorum* family and seeds of *Lepidium sativum* (purchased from TORAF, Poland) seeds. These objects can be kept in an anhydrous state over a wide temperature range and for a long time. After providing the culture medium and appropriate external conditions, they begin their growth. Detailed data on each of the cultures are provided in Table 3.

**Table 3:** Fungi and seed culturing parameters.

	Fungi (*Fusarium culmorum*)	Seed (*Lepidium sativum*)
Culturing gas mixture	Air at atmospheric pressure	Air at atmospheric pressure
Optimal culture temperature [°C]	27—28	15—30
Culture temperature tolerance [°C]	10–35	
Medium	Water	
Culturing time [days]	Approx. 15–30	Approx. 5–14
Lightning	White	
Culture success criterion	Mycelium growth	Seed germination

### Lab-payload elements

The operation of three biological experiments simultaneously was planned. Each of them had to be provided with a dedicated lab-chip for cells/seed, appropriate temperature for the cultivation, proper dosing of the medium, lighting, and signal detection in the form of recorded photos.

#### Lab-chips

**Fig. 3:**
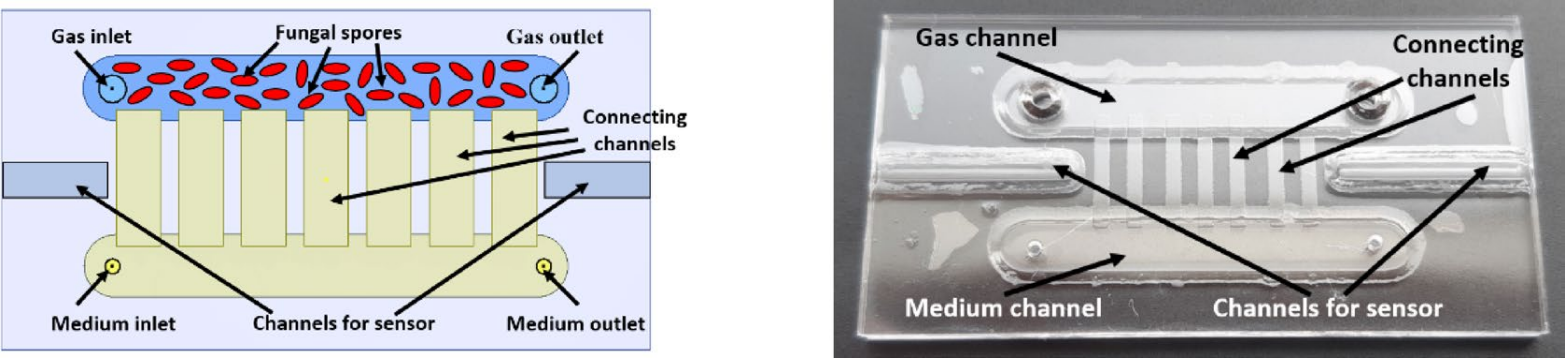
Design of the lab-chip for fungi cultivation: scheme (left), ready-to-use structure (right).

Dedicated lab-on-chips enabling the culture of fungi/seed were designed and fabricated. The layout topology was chosen to ensure optimal development of each of the cultures. Borosilicate glass (Borofloat33, Schott) was chosen as the material for the construction of lab-chip for fungi culture. The bottom glass substrate has two channels, the dimensions of a single channel are 3.5 mm × 30 mm, and the depth is 550 μm. In one of them, nourishment flows (medium channel), while in the other, culturing takes place (gas channel). Holes in two sizes were created in the top substrate. Smaller ones (ø 1 mm), allowing water to be introduced and removed from the flow channel, and larger ones (ø 2 mm), which ensure free diffusion of gas in the culturing channel. The top substrate also has a matrix of seven shallower microchannels (connecting channels), each measuring 8.8 mm × 1 mm and 20 μm deep (Fig. 3). They connect the medium and gas channels, providing the gas (culturing) channel with humidity appropriate for the growth of the spores. In the bottom and top substrate two channels were made for placing temperature sensors. This design allows for direct temperature measurement within the lab-chip. The glass lab-chip substrates had standard dimensions of 50 mm × 25 mm × 1.1 mm. The structuring of glass substrates was carried out using wet etching, mechanical drilling, and low-temperature bonding processes (Fig. 3)^35^. The fabricated lab-chip made meets the requirements for cultivating fungal spores, including ensuring a constant supply of air and maintaining the appropriate level of humidity.

**Fig. 4:**
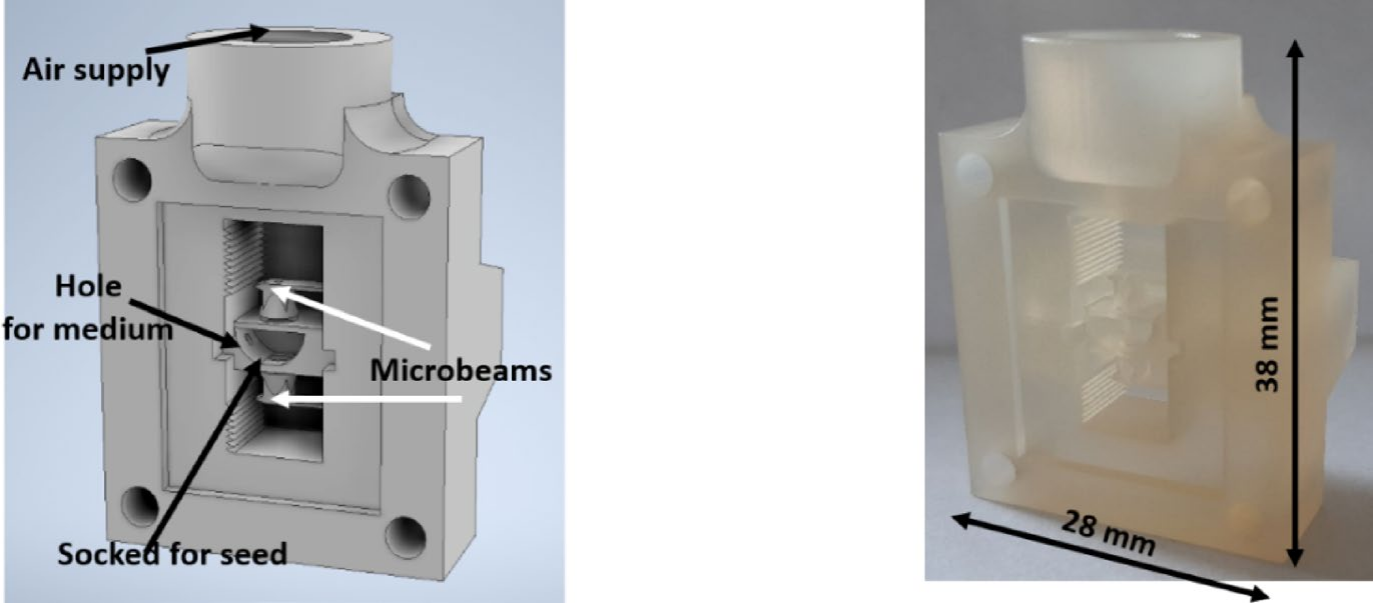
Design of the micropot for seed cultivation: scheme (left), 3D printed structure (right).

The micropot used for growing cress seeds has a socket in its central part for placing the seed and four holes extending from it. The side holes allowed for the exchange of the culture medium. The upper and lower holes of the socket allow the root and stem of the growing seed to enter the flexible microbeams*, enabling* the determination of the seed’s growth potential^34^. The upper, large opening allows gas exchange (Fig. 4). The micropot was made with InkJet 3D printing technology (ProJet 3510 HD, 3D Systems). VisiJet M3 Crystal light-curing resin was used as the building material and VisiJet S300 paraffin material as a supporting material. After postprocessing, a ready-made micropot was obtained (Fig. 4).

For fungi and seed cultures, experiments in simulated microgravity were carried out using of a Rotating Wall Vessel—RWV and Random Positioning Machine—RPM^34,35^. It could be seen that the fungal spores grew better in the simulated microgravity. In contrast, the cress seed had a problem with proper development. Simulated microgravity conditions could have disrupted the growth of the plant. In typical conditions, geotropism is a mechanism that is responsible for plant response to gravity. Thus, roots can grow downward (positive geotropism), whereas stems can grow upward (negative geotropism). Our experiments have shown that even simulated absence of gravity force can notably influence the plant growth. It is probable that disturbances in specialized cells known as statocytes, which are responsible for sensing the gravity vector during the early stages of germination, could have occurred.

#### Dosing system

**Fig. 5:**
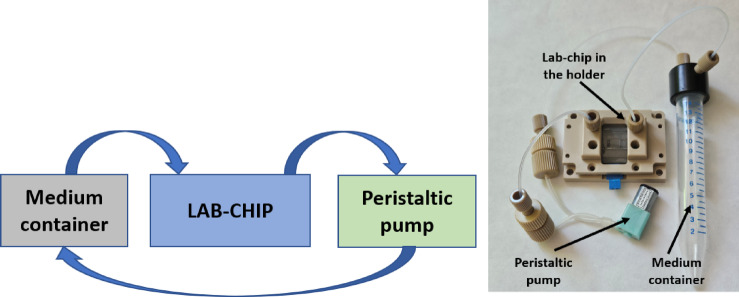
Dosing system: scheme (left), real view (right).

An essential component of the system was the dosing of the culturing medium, which operated in microgravity conditions. It used a medium container (Eppendorf, Germany) and a peristaltic pump (Takasago, Japan). Based on our previous works^35^, it was defined that closed medium circulation is appropriate for the research. Moreover, such a configuration is considered less complex and space-saving, thus we used a similar approach herein. Flow rate, as well as medium volume needed for the experiment were determined experimentally. On that basis, it was indicated that a medium container capacity of 15 ml is sufficient for grain (culture time up to 14 days) and fungi cultivation (culture time up to 30 days). For the fungi, a pulse operation was set at 200 µl/min for 60 s every 10 h, whereas for the grain, it was 500 µl/min for 25 s every 12 h (Fig. 5).

#### Detection system

**Fig. 6:**
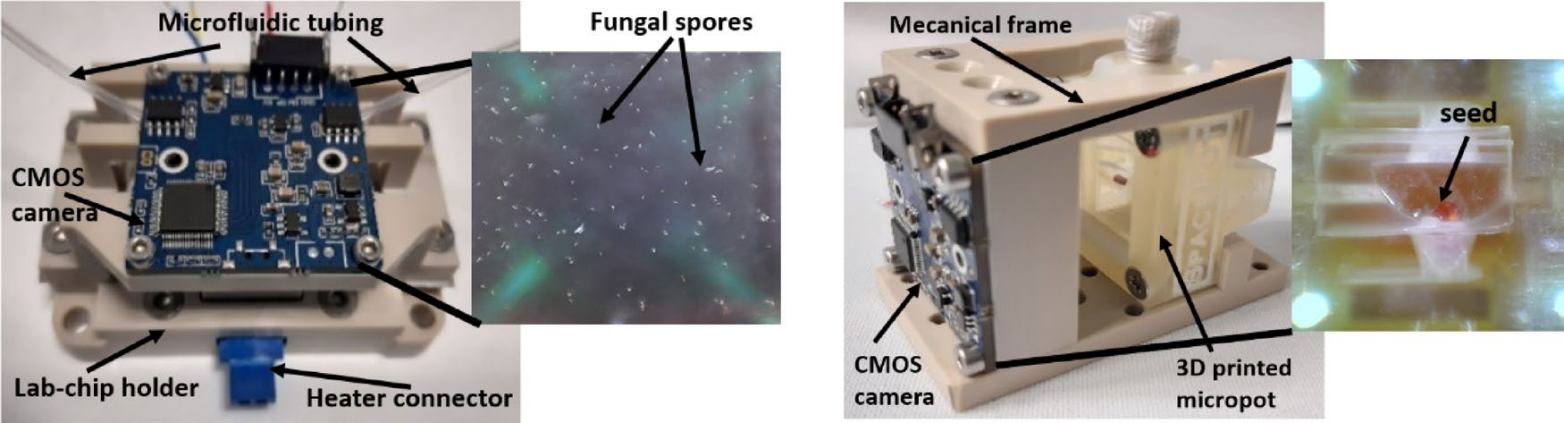
Detection system integrated with lab-chip for fungi cultivation (left), micropot for seed cultivation (right) and examples of photographed obtained.

In order to parametrically evaluate the culture development, an optical observation system was proposed allowing for image acquisition. The key requirement for the system was the ability to obtain significant magnification and high image resolution, as well as the ability to control image sharpening (due to the sample growth). A miniature CMOS camera ELP-USB8MP02G with a Sony IMX179 8MPx matrix (3264 × 2448 pixels, pixel size 1.4 µm) with USB 2.0 interface and specially selected electromagnetically controlled focus from the Logitech C615 Portable HD Webcam were used (Fig. 6). The samples were observed in reflection mode. To illuminate the regions of interest, LED diodes were used. It was important to mount the camera with focus and lighting just above the lab-chip surface, so that the system could withstand the 10G overload generated during rocket launch. The detection system was universal and allowed the observation of microscale objects, with dimensions of 8 µm (fungi), as well as larger ones, such as cress seed.

#### Temperature control system (TCS) and payload service (PS) module

**Fig. 7:**
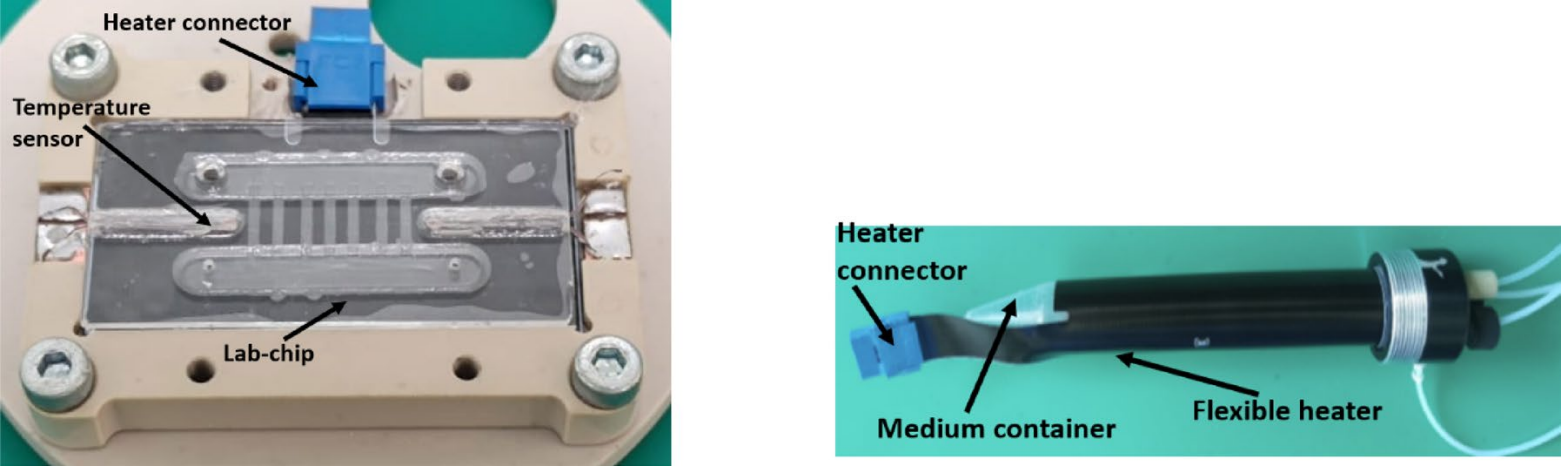
Flex PCB heater: mounted under the lab-chip (left), integrated with medium container (right).

**Fig. 8:**
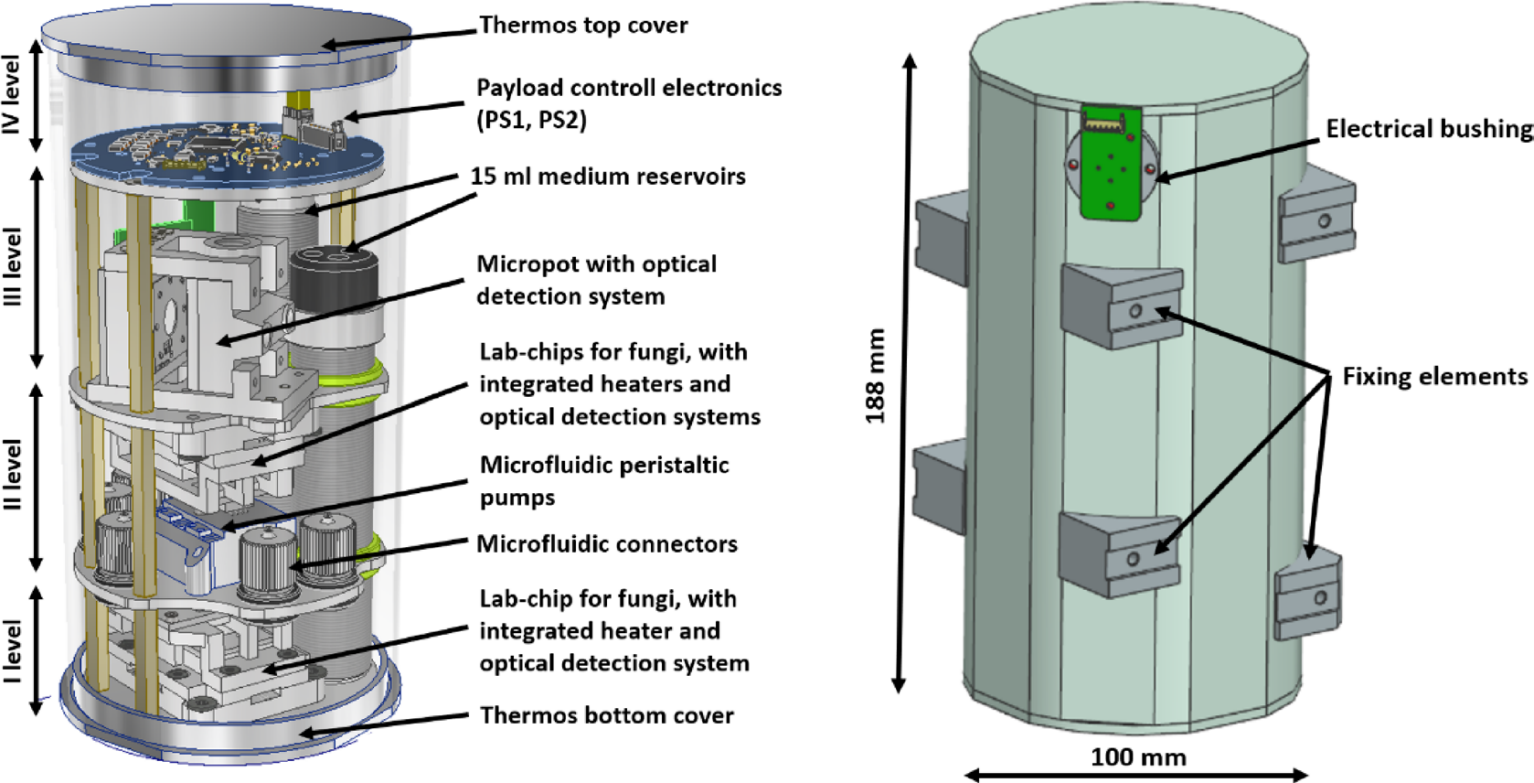
3D model of the lab-payload: insert (left), packaging (thermos - right).

The Temperature Control System (TCS) is responsible for maintaining the temperature of the biological sample and its medium, which require reaching temperatures in the range of 20–30 °C, depending on the experiment. TCS was implemented using thin resistive heating circuits (Flex PCBs) integrated with each lab-chip (Fig. 7) and medium container (Fig. 8). Accompanying embedded temperature sensors (NTC 10 kΩ in lab-chips and PT100 on medium containers) enabled self-regulating temperature control in the PID feedback loop.

Apart from the culturing systems, additional environmental measurements in the lab-payload volume were carried out by using an integrated sensor of pressure, temperature and humidity—BME280 (Bosh), NTC thermistors 10 kΩ (TDK) and radiation dose sensors RADFET (Varadis). The BME280 modules and RADFETs were placed directly on the Payload Service PCB (described below), while the NTC sensors were soldered “on wires” and placed close to the culturing systems, but beyond them.

Inside the payload there was also an internal electronic circuit PS (Payload Service). It was responsible for autonomously managing the cultivation of three biological samples. It carried out cyclic dosing of nutrient solution into lab-chips and micropot, measured and controlled the temperature of fungi cultures and water tanks for fungi and seed, acquired and recorded photos of fungi and seed, including controlling image focus and lighting, and performed on-demand measurements of pressure values, humidity, temperature, and accumulated radiation dose inside the lab—payload. Telecommand and telemetry, as well as payload data exchange between PS and the satellite communication module, were performed via a CAN interface using the CSP protocol.

### Lab-payload construction

The mechanical structure of the lab-payload secured and properly positioned all elements in the cylindrical packaging. The internal structure consists of four circular disks (levels), connected to each other at an appropriate distance. On the first level and the lower surface of the third level, glass lab-chips in packaging with heating and optical detection systems were placed. On the second level, all IDEX microfluidic connectors and two pumps used for fungi cultivation were placed. On the upper surface of the third level a micropot with an optical detection system and a fluid supply pump for the seed were located. In the second and third levels, there were two dedicated fluid tanks, one for fungi and one for seed. The fourth level housed the PS1 and PS2 modules (Fig. 8). The structural elements were made of PEEK (polyetheretherketone). This material was selected, among others, due to its low weight, good strength and insulation parameters, as well as biocompatibility and low outgassing coefficients.

The packaging (thermos) was used as a mechanical, radiation, and thermal cover. Under vacuum conditions, it kept air inside at a pressure of approximately 1000 hPa. The construction material was aluminum. The packaging body was a pipe with an outer diameter of 100 mm and a height of 188 mm. The pipe was closed at the top and bottom with covers. There was an electrical bushing on the side wall enabling connection to the satellite base. The thermos had four cuts on the external surface enabling installation in a CubeSat structure (Fig. 8).

### LabSat satellite with payload, integration

The lab-payload, described above, was the main component of the LabSat bio-nanosatellite. Lab-payload performed a task directly related to the mission goal in orbit. The satellite platform enabled the proper functioning of the lab-payload in LEO and its operation from Earth using a radio link. The lab-payload was integrated with a satellite platform equipped with a set of basic electronic modules, i.e. ADCS (Attitude Determination and Control System) position determination and control system, an Auxiliary Power Supply (APS) which provided an appropriate voltage range for the lab-payload and a CM LF (Communication Module Low Frequency) radio module.

**Fig. 9:**
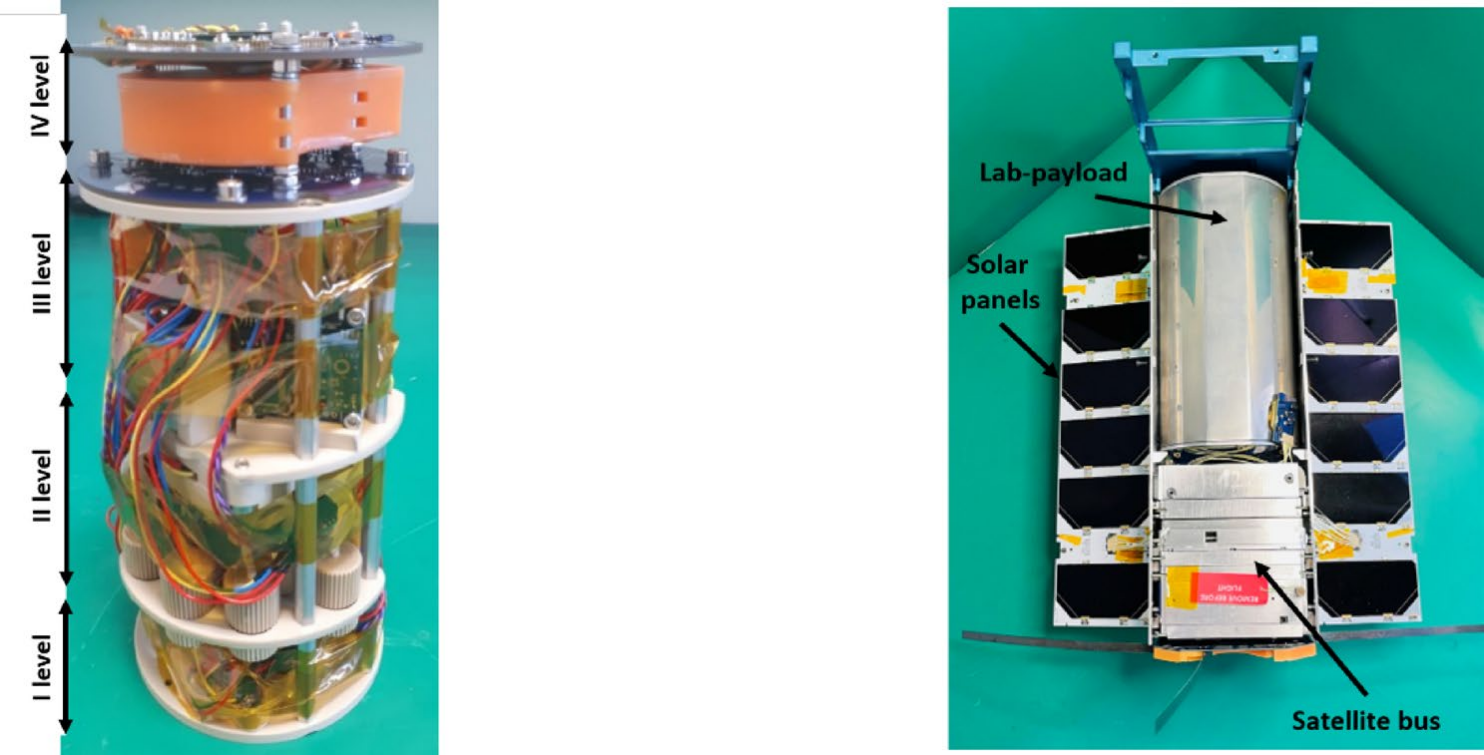
Integrated lab-payload (left) and lab-payload in the LabSat satellite (right).

All components of the lab-payload were integrated (Fig. 9) and mounted inside the thermos. The resulting engineering model (EM) underwent thermal, thermal-vacuum (TVAC), random vibration (RVT), and electromagnetic compatibility (EMC) tests. No anomalies were observed. After some design improvements, a flight model (FM) of the lab-payload has been developed, which again successfully went through the thermal and thermal-vacuum verification and has been integrated with the proto-flight model (PFM) of the satellite platform. The complete LabSat satellite successfully passed the RVT, qualifying it for rocket flight (Fig. 9).

### Mission

**Fig. 10:**
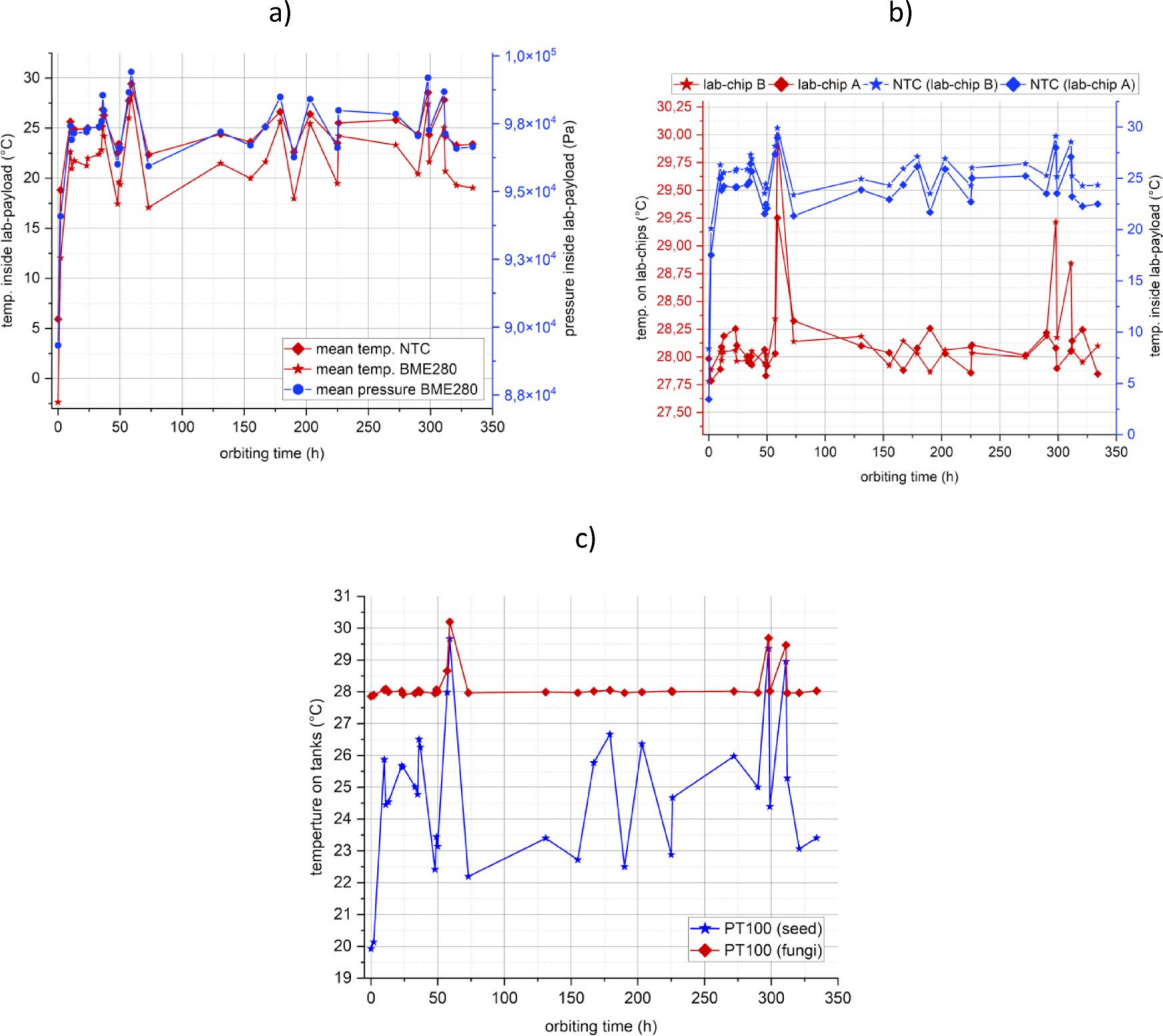
Measurements from the orbit: (**a**) average pressure and temperature in the lab—payload volume; (**b**) average temperatures on the lab-chips and temperatures from individual NTCs placed in the lab-payload volume near the lab-chips (as a reference); (**c**) temperatures measured on medium tanks; time “0 h” corresponds with the moment of first contact with the satellite.

In January 2022 LabSat was launched aboard a Falcon 9 rocket. It was part of the shared Transporter—3 mission. After 15 days orbiting on the Satellite Vehicle Carrier (SVC) provided by D-Orbit, the nanosatellite was removed from the deployer and placed on orbit with the assumed parameters (altitude: approx. 550 km, inclination: 97.5°). Communication was established roughly one hour after deployment and the parameters of all satellite modules have been defined. After achieving of the correct conditions, the heating and dosing medium modules were activated and biological experiments were started and lasted 14 days. During the mission, various parameters were monitored, including pressure and temperature inside the lab-payload (Fig. 10a), on individual lab-chips for fungal cultivation (Fig. 10b), and on the medium tanks (Fig. 10c).The obtained temperature values were consistent with expectations and enabled proper cultivation (as defined in Table 3). The hermeticity of the thermos was also confirmed (Fig. 10a), and the indicated pressure changes were related to temperature fluctuations inside lab-payload, caused by variations in sunlight exposure as the satellite moves along its orbit.

**Fig. 11:**
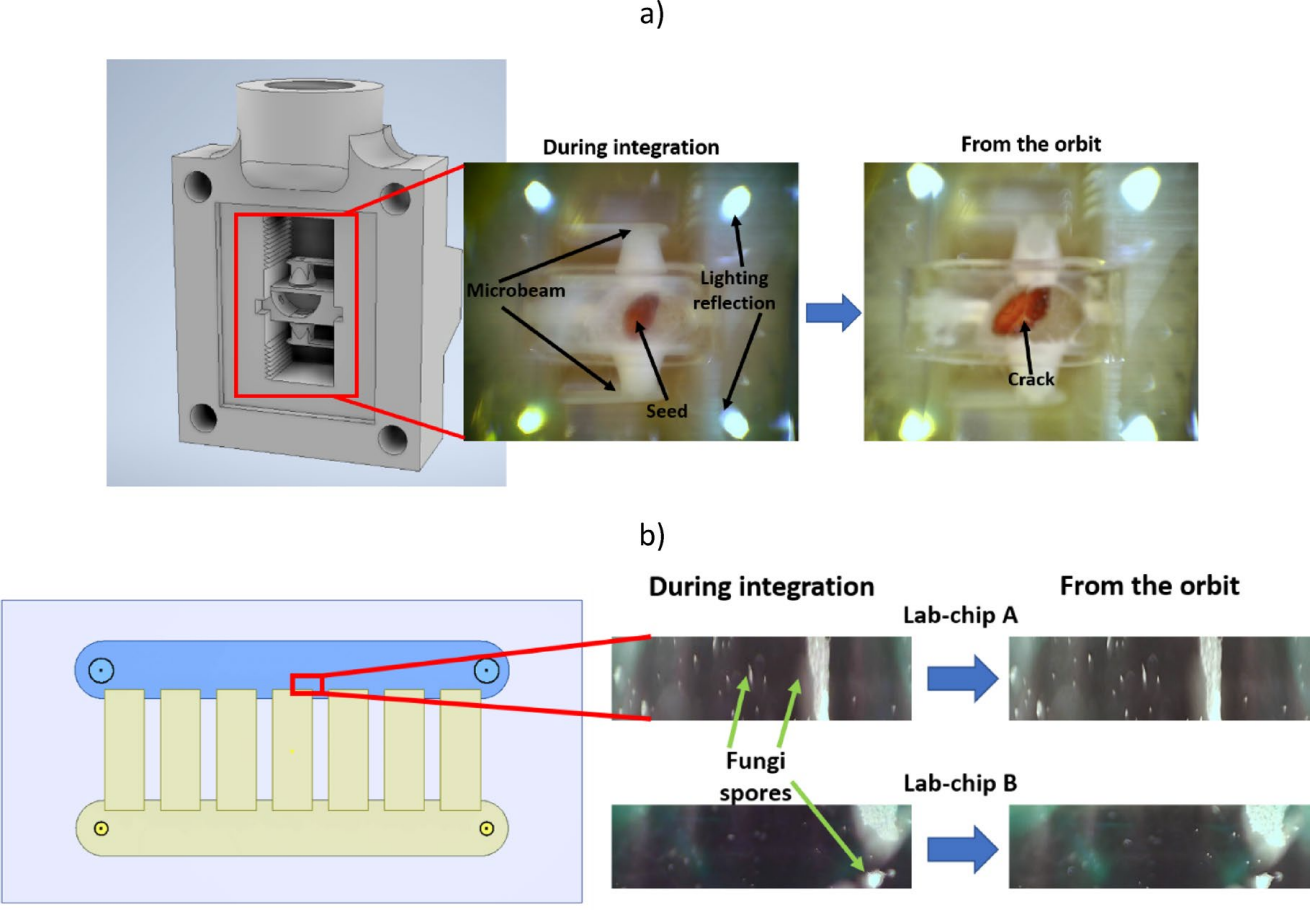
Course of the experiment: (**a**) cress seed in a micropot during the experiment; (**b**) fungi spores in two lab-chip during the experiment.

The photo documentation was downloaded. We obtained an image of the grain, which shows swelling and cracking of the husk, indicating the appropriate supply of the medium and the correct initial stage of plant growth (Fig. 11a). Partial photos of two lab-chips A and B in which fungi cultures were carried out were also obtained (Fig. 11b). The images were taken approximately 36 h after the start of the experiment in orbit. The detection system we prepared with controlled focus change remained undamaged, and the images obtained confirm the maintenance of the required parameters of the detection system.

Due to communication difficulties with the nanosatellite bus and, ultimately, the loss of contact on the fourteenth day of the mission, it was not possible to obtain more experimental data. Thus, tracking the further growth of the cress grain and fungi cultures in microgravity conditions was stopped. However, the obtained data appear sufficient to confirm the reliable operation of the developed lab–payload components.

## Conclusion

Small scale CubeSat satellites used for biomedical research in microgravity are important instrumentation which further development can bring notable benefits in space science and exploration. Continuous exploitation of this domain of science will allow us to safely plan for future missions both to the Moon and to Mars, assuming human participation. The use of nanosatellites makes it possible to study the influence of several space-related factors simultaneously, in a single mission, in real space conditions. It is also possible to study various biological objects in a consistent and repeatable way. Such missions are relatively low-cost and can be operated in a remotely controlled way, with minimal human participation. Cooperation in conducting this type of mission leads to the rapid development of individual domains of science, which at the same time contributes to the creation of many solutions that can be applied on Earth.

Within the presented article, the full functionality of the autonomous, miniature research station, called the lab-payload, was confirmed. The lab-payload allowed research to be conducted on two different biological objects (*Fusarium culmorum, Lepidium sativum*) during one nanosatellite mission, which was the second such case in the world. The laboratory payload proposed here provides autonomous activation and experimentation on LEO. The correct operation of the medium dosing, heating, and detection systems was confirmed. Moreover, for the first time, two different types of lab-chips for cell culturing (glass lab-chip and 3D printed micropot) were provided and successfully tested in a single mission. As presented in the paper, interesting biological results were also achieved showing swelling and cracking of the cress seed husk in microgravity conditions. The time of securing and storing biological samples in lab-chips was also important, from the moment of integration with the satellite to the launch of the rocket (38 days), then placing the satellite in orbit (15 days) and the duration of the experiment itself (14 days). Despite communication problems during the mission, it was possible to confirm the correct operation of the lab-payload components, which allows us to conclude that these solutions can be used and/or adapted for subsequent satellite missions. Undoubtedly, the results of the LabSat mission described in this paper constitute a substantial building block in developing the field of biological satellite research programs.

To date, together with LabSat described in this article, 11 biological nanosatellite missions have been sent. The outcomes of such missions can significantly contribute to the understanding of biological processes under space conditions, thereby enabling the assessment of both the detrimental and beneficial effects of space exposure on living matter. As with any such undertaking, these missions entail numerous challenges and the need to overcome various limitations.

In future work of this kind, the authors propose several recommendations that may facilitate the development of orbital laboratories. Microfluidic systems, including lab-chip platforms, are considered the most advantageous and efficient for conducting experiments involving living organisms in space environments. However, it is worth considering the use of emerging technologies such as 3D printing for the fabrication of connectors, adapters, valves, and regulators, which could enable more compact system architectures.

The proposed method for monitoring and parameterizing culture development based on image analysis is broadly applicable. Nevertheless, previously employed techniques, such as colorimetry and spectroscopy, have proven equally effective—particularly in scenarios involving multiple culture targets or where the expected biological response (e.g., metabolic activity) can be precisely defined.

The temperature control system (TCS) developed for this mission was deemed adequate for maintaining appropriate thermal conditions for the presented experiment. However, for future projects, it is advisable to validate the TCS within the final configuration (i.e., in the payload and satellite structure) using TVAC (Thermal Vacuum Chamber) testing, which more accurately simulates the thermo-vacuum environment of orbit.

It would also be beneficial for the payload support electronics module (PS) to perform continuous environmental data logging, allowing for a comprehensive interpretation of culture results. To this end, employing a higher-bandwidth UHF radio module would be necessary to ensure improved data transmission and communication capabilities.

Additionally, the implementation of flexible Kapton tape wiring with edge connectors is recommended, as it could substantially reduce both integration time and complexity. With regard to the assembly of laboratory subsystems, the development of modular, pre-assembled and repeatably integrable components is encouraged, which would streamline the integration process of the entire device.

Finally, it is important to emphasize that few biological systems are naturally capable of surviving extended periods of metabolic suspension prior to launch. Therefore, ongoing research into preservation methods for biological payloads, as well as advancements in satellite launch strategies, is essential.

## Data Availability

The authors declare that the data supporting the findings of this study are available within the paper.

## References

[1] Clément, G., Slenzka, K. Fundamentals of space biology : research on cells, animals, and plants in space, (2006), Springer Science + Business Media, 10.1007/0-387-37940-1

[2] Herranz, R. et al. Ground-based facilities for simulation of microgravity: Organism-specific recommendations for their use, and recommended terminology. *Astrobiology* **13**, 1–17. 10.1089/ast.2012.0876 (2013). 23252378 10.1089/ast.2012.0876 PMC3549630

[3] Zhang, S. et al. Simulated microgravity using a rotary culture system compromises the in vitro development of mouse preantral follicles. *PLoS ONE*10.1371/journal.pone.0151062 (2016). 26963099 10.1371/journal.pone.0151062 PMC4786255

[4] Beysens, D. A. van Loon, J. J. W. A. Generation and applications of extra-terrestrial environments on Earth, (2015), River Publishers doi.org/10.13052/rp-9788793237544

[5] Harland, D. M., Catchpole, J. E. Creating the International Space Station, (2002), Springer-Praxis

[6] Ohnishi, T. Life science experiments performed in space in the ISS/Kibo facility and future research plans. *J. Radiat. Res.* **57**, 41–46. 10.1093/jrr/rrw020 (2016). 10.1093/jrr/rrw020 PMC4990110 27130692

[7] Cappelletti, Ch., Battistini, S. & Malphrus, B. K. Cubesat handbook from mission design to operations. *Elsevier*10.1016/C2018-0-02366-X (2021).

[8] Poghosyan, A. et al. CubeSat evolution: Analyzing CubeSat capabilities for conducting science missions. *Prog. Aerosp. Sci.* **88**, 59–83. 10.1016/j.paerosci.2016.11.002 (2017).

[9] Krakos, A. Lab-on-chip technologies for space research — current trends and prospects. *Microchim Acta* **191**, 31. 10.1007/s00604-023-06084-4 (2024). 10.1007/s00604-023-06084-4 PMC10721686 38095809

[10] Kitts, C. et al, Flight Results from the GeneSat-1 Biological Microsatellite Mission, The Proceedings of the 21^st^ Annual AIAA/USU Conference on Small Satellites, 2007

[11] Ricco, A. J. et al., PharmaSat: drug dose response in microgravity from a free-flying integrated biofluidic/optical culture-and-analysis satellite, Proceedings Microfluidics, BioMEMS, and Medical Microsystems IX, 2011, vol. 7929, 10.1117/12.881082

[12] Ehrenfreund, P. et al. The O/OREOS mission – Astobiology in Earth orbit. *Acta Astronaut.* **93**, 93. 10.1016/j.actaastro.2012.09.009 (2014).

[13] Bramall, N. E. The development of the space environment viability of organics (SEVO) experiment aboard the Organism/organic exposure to orbital stresses (O/OREOS) satellite. *Planetary Space Sci.*10.1016/j.pss.2011.06.014 (2012).

[14] Park, J. et al. An autonomous lab on a chip for space flight calibration of gravity-induced transcellular calcium polarization in single-cell fern spores. *Lab Chip* **17**(6), 1095–1103. 10.1039/c6lc01370h (2017). 28205656 10.1039/c6lc01370h

[15] SporeSat (SpaceX-3), https://www.nasa.gov/ames/space-biosciences/sporesat-spacex-3/ (accessed August 1, 2024)

[16] Padgen, M. R. et al. EcAMSat spaceflight measurements of the role of σs in antibiotic resistance of stationary phase Escherichia coli in microgravity. *Life Sci. Space Res. (Amst).*10.1016/j.lssr.2019.10.007 (2020). 31987476 10.1016/j.lssr.2019.10.007

[17] EcAMSat Satellite, https://www.nanosats.eu/sat/ecamsat, (accessed August 1, 2024)

[18] Amselem, S. Remote controlled autonomous microgravity lab platforms for drug research in space. *Pharm Res.*10.1007/s11095-019-2703-7 (2019). 31741058 10.1007/s11095-019-2703-7

[19] DIDO-3 Satellites, https://www.nanosats.eu/sat/dido, (accessed August 1, 2024)

[20] Padgen, M. R. et al. BioSentinel: A biofluidic nanosatellite monitoring microbial growth and activity in deep space. *Astrobiology*10.1089/ast.2020.2305 (2021). 33601926 10.1089/ast.2020.2305

[21] Tieze, S. M. et al. BioSentinel: A biological CubeSat for deep space exploration. *Astrobiology*10.1089/ast.2019.2068 (2023). 10.1089/ast.2019.2068 PMC10254969 32282239

[22] Obreque, E. et al, The First Chilean Satellite Swarm: Approach and Lessons Learned The Proceedings of the 37^th^ Annual AIAA/USU Conference on Small Satellites, 2023

[23] Marzioli, P. et al, Autonomous cultivation system for nano platforms: the GreenCube mission, Proceedings of the 73^rd^ International Astronautical Congress (IAC), 2023

[24] Marzioli, P. et al, The GreenCube CubeSat mission: Development and Qualification of an autonomous Microgreens Cultivation System and demonstration of CubeSat propulsion in MEO, Proceedings of the 72^nd^ International Astronautical Congress (IAC), 2022

[25] Carletta, S. et al, A nanosatellite operating in the Van Allen belt: The lessons learned from the AstroBio CubeSat mission, Proceedings of the 73^rd^ International Astronautical Congress (IAC), 2023

[26] Nascetti, A. et al, In-orbit Characterization of a Lab-on-Chip Payload with Integrated Thin-Film Photosensors for Chemiluminescent Immunoassays aboard the AstroBio CubeSat Mission, Proceedings of 9th International Workshop on Advances in Sensors and Interfaces, 2023

[27] Calabria, D. et al. AstroBio-CubeSat: A lab-in-space for chemiluminescence-based astrobiology experiments. *Biosensors Bioelectron.*10.1016/j.bios.2023.115110 (2023). 10.1016/j.bios.2023.115110 36750012

[28] Krakos, A. et al. Microfluidic-assisted human cancer cells culturing platform for space biology applications. *Sensors* **22**, 6183. 10.3390/s22166183 (2022). 36015950 10.3390/s22166183 PMC9414851

[29] Ley, W., Wittmann, K., Hallmann, W. Handbook of space technology, (2009), Wiley, 10.1002/9780470742433

[30] Horneck, G., Rettberg, P. Complete course in astrobiology, (2008), Wiley

[31] SatRev Inc., „Universal Platform - Interface Control Document Rev. 2.1”, 23 June 2021, technical documentation, available by submitting a request via the form on the company’s website, Wrocław, Poland: Rev. 2.1. Access: 28 May 2025. [Online]. Available at: https://www.satrev.space/contact.

[32] CubeSat Design Specification Rev. 14.1 The CubeSat Program, Cal Poly SLO CubeSat Design Specification Cal Poly-San Luis Obispo, CA Document Classification X Public Domain

[33] eCFR, (n.d.). https://www.ecfr.gov/current/title-49/subtitle-B/chapter-I/subchapter-C/part-172/subpart-B/section-172.101 (accessed August 29, 2022)

[34] Kawa, B. et al. Nanosatellite payload for research on seed germination in a 3D Printed Micropot. *Sensors*10.3390/s23041974 (2023). 36850572 10.3390/s23041974 PMC9962095

[35] Krakos, A. et al. Lab-on-chip culturing system for fungi—Towards Nanosatellite missions. *Appl. Sci.*10.3390/app122010627 (2022).

